# Recent Advances in Ultrasound Breast Imaging: From Industry to Clinical Practice

**DOI:** 10.3390/diagnostics13050980

**Published:** 2023-03-04

**Authors:** Orlando Catalano, Roberta Fusco, Federica De Muzio, Igino Simonetti, Pierpaolo Palumbo, Federico Bruno, Alessandra Borgheresi, Andrea Agostini, Michela Gabelloni, Carlo Varelli, Antonio Barile, Andrea Giovagnoni, Nicoletta Gandolfo, Vittorio Miele, Vincenza Granata

**Affiliations:** 1 Department of Radiology, Istituto Diagnostico Varelli, 80126 Naples, Italy; 2 Medical Oncology Division, Igea SpA, 80013 Naples, Italy; 3 Department of Medicine and Health Sciences “V. Tiberio”, University of Molise, 86100 Campobasso, Italy; 4 Division of Radiology, “Istituto Nazionale Tumori IRCCS Fondazione Pascale-IRCCS di Napoli”, 80131 Naples, Italy; 5 Department of Diagnostic Imaging, Area of Cardiovascular and Interventional Imaging, Abruzzo Health Unit 1, 67100 L’Aquila, Italy; 6 Italian Society of Medical and Interventional Radiology (SIRM), SIRM Foundation, 20122 Milan, Italy; 7 Department of Clinical, Special and Dental Sciences, University Politecnica delle Marche, 60126 Ancona, Italy; 8 Department of Radiology, University Hospital “Azienda Ospedaliera Universitaria delle Marche”, 60126 Ancona, Italy; 9 Department of Translational Research, Diagnostic and Interventional Radiology, University of Pisa, 56126 Pisa, Italy; 10 Department of Applied Clinical Sciences and Biotechnology, University of L’Aquila, 67100 L’Aquila, Italy; 11 Diagnostic Imaging Department, Villa Scassi Hospital-ASL 3, Corso Scassi 1, 16149 Genoa, Italy; 12 Department of Emergency Radiology, Careggi University Hospital, 50134 Florence, Italy

**Keywords:** ultrasound, breast imaging, clinical practice

## Abstract

Breast ultrasound (US) has undergone dramatic technological improvement through recent decades, moving from a low spatial resolution, grayscale-limited technique to a highly performing, multiparametric modality. In this review, we first focus on the spectrum of technical tools that have become commercially available, including new microvasculature imaging modalities, high-frequency transducers, extended field-of-view scanning, elastography, contrast-enhanced US, MicroPure, 3D US, automated US, S-Detect, nomograms, images fusion, and virtual navigation. In the subsequent section, we discuss the broadened current application of US in breast clinical scenarios, distinguishing among primary US, complementary US, and second-look US. Finally, we mention the still ongoing limitations and the challenging aspects of breast US.

## 1. Introduction—Where Do We Come from?

In 1977, Dr. Dodd wrote: “The spatial resolution presently obtainable in ultrasonograms is inadequate for the detection of subclinical [breast] cancer” [1]. Later still in 1983, Sickles and co-workers claimed: “These data indicate that sonography is not an acceptable substitute for mammography in the detection and diagnosis of breast cancer” [2]. For many years, the main use of breast ultrasound (US) was for differentiating cystic lesions from solid lesions [3]. Consequently, US has become an integral component of the diagnostic work-up of breast abnormalities only in the last two decades [4,5,6,7,8]. The seminal article on using US for differential diagnosis was only published in 1995 [9], and the first BI-RADS atlas addressing the issue of US, in addition to mammography, was only published in 2003 [10]. The dramatic increase in spatial resolution is the main explanation for the advancing role of US. The application of tissue harmonics, spatial compound imaging, and speckle reduction techniques further refined the images [3,11]. Appropriate scanner settings and optimized scanning methodologies are also mandatory for an up-to-date US examination of the breast.

Technological advances now allow a comprehensive US diagnosis, management, and treatment of breast abnormalities. In this review, we discuss the technical revolution that happened over the years and point out the consequent clinical revolution that has occurred in daily practice.

## 2. Technological Developments—What the Industry Has Made Available to Us

### 2.1. Conventional Doppler Techniques and New Microvasculature Imaging Techniques

**Figure 1:**
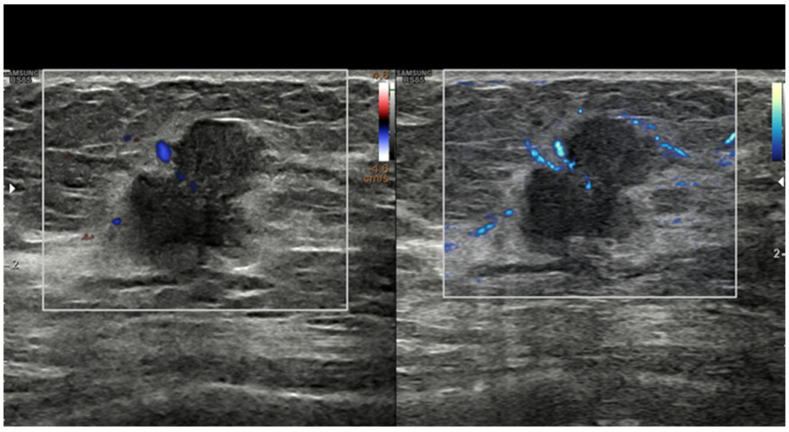
Breast invasive ductal carcinoma. Vascularization as assessed with power Doppler (**left**) and with MV-Flow (**right**). Recently developed techniques such as MV-Flow are more sensitive to slow flows and offer a better display of tumor vessels.

Cancer growth is based on neoangiogenesis, i.e., the tumor-induced development of a vascular network. Detecting these flow signals and assessing their characteristics in terms of number, distribution, and appearance are consequently of paramount importance in tumor characterization and monitorization [12,13,14,15,16,17,18,19,20,21,22,23,24,25,26,27,28]. Even if appropriately set to identify small, low-flow vessels, conventional Doppler techniques, including color Doppler and power Doppler, have a limited sensitivity. In recent years, almost all companies have developed filtered techniques capable of working at a higher frame rate and consequently detecting tiny intra- and peri-tumoral flow signals. With these new software facilities, the background and tissue motion artefacts are suppressed and US sensitivity, spatial resolution, and temporal resolution are significantly improved [29,30,31,32,33,34,35,36,37,38]. Some companies have also developed systems capable of quantifying the number of colored pixels within the box, thus quantifying the flow intensity [39]. If a new microvascular tool is available on the scanner, we encourage users to refrain from still using conventional Doppler modalities for breast imaging and to employ more advanced techniques (Figure 1).

### 2.2. High-Frequency Transducers

**Figure 2:**
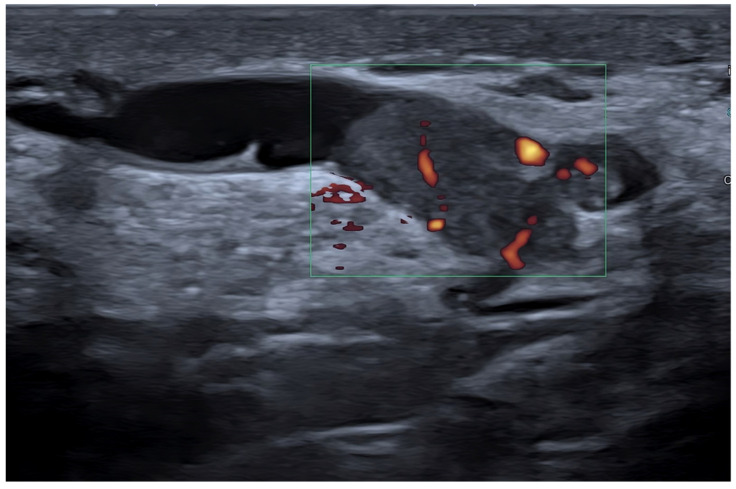
Breast intraductal papilloma. Detailed morpho-structural assessment working with a high-frequency transducer (22 MHz). Power Doppler detection of tumor vessels.

The transmission frequencies employed to scan breasts have increased through the years, moving from 7.5 MHz initially to 10–12 MHz and currently to 13–18 MHz. High-frequency transducers provide increased axial and soft tissue resolution, permitting improved differentiation of subtle shades of gray, margin resolution, and lesion conspicuity in the background of normal breast parenchyma [11]. Current transducers have a broad band of frequencies, allowing the operator to choose the most appropriate one in relation to the size of the given breast and based on the depth of the area of interest within it. However, most US companies now sell probes reaching frequencies up to 22–24 MHz. These transducers have been developed to study the skin, but there are a number of circumstances where they can be adopted to investigate the breast. These include skin abnormalities of the breast and axilla, nipple–areolar complex abnormalities, very small-size breasts, superficial areas in any size breasts, prepuberal breasts, male breasts, breast parenchyma abnormalities in subjects with implants, post-mastectomy chest wall, and intraoperative breast sonography [32] (Figure 2).

### 2.3. Extended Field-of-View Scanning

US has a limited field of view (FOV), not allowing to display, in a single scan, a breast area larger than the probe footprint itself [11]. Extended FOV systems allow to partially encompass this limitation. Starting from a real-time translational movement of the probe over the skin, the software can continuously compare the position and create a large 2D image, without any loss in terms of spatial resolution.

**Figure 3:**
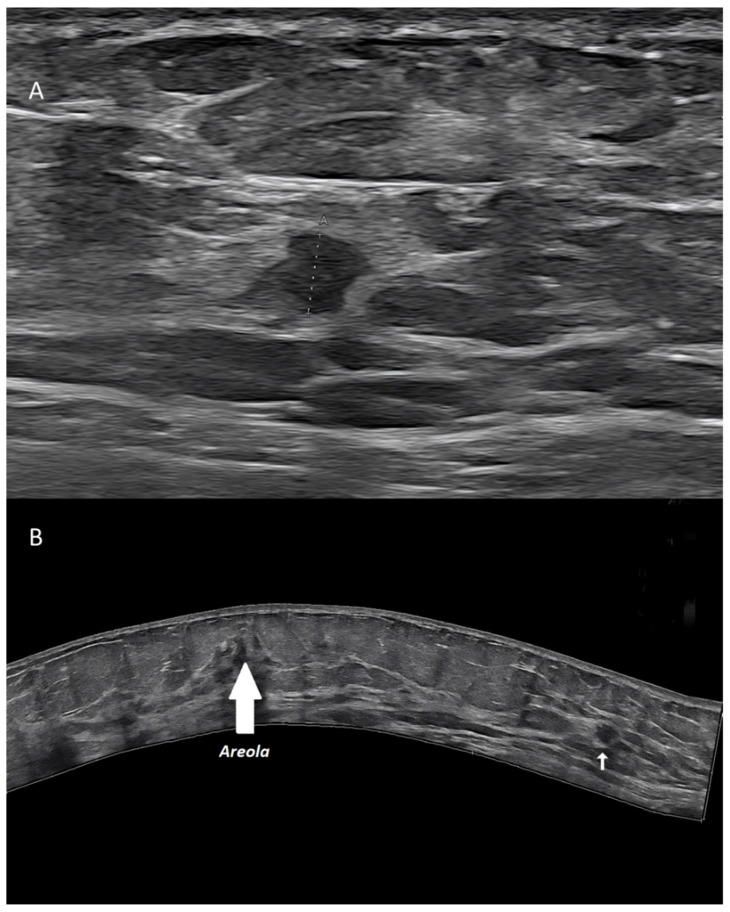
Invasive ductal carcinoma of the breast. (**A**) US scan showing a 4-mm nodule (calipers). (**B**) Extended FOV scan displaying the nodule (arrow) within the whole breast.

Extended FOV scans allow to display and measure large breast lesions. Additionally, it becomes possible to show the spatial relationship and the distance between a lesion and some anatomic landmarks, such as the nipple, or between multiple lesions, as for multifocal/multicentric cancer [13] (Figure 3). Images of the whole implant can be obtained, being particularly useful for plastic surgeons.

### 2.4. Elastography

US elastography measures small tissue motions due to pressure forces, i.e., the viscoelastic properties of breast abnormalities [40]. Current techniques include strain elastography, based on the manual pressure from the operator; acoustic radiation force impulse imaging (ARFI); point shear-wave elastography; and 2D/3D shear-wave elastography, based on the changes provoked from the focused ultrasounds themselves. Based on the different techniques, qualitative (subjective scoring), semi-quantitative (strain-to-fat ratio), and/or quantitative data can be obtained [7,8,37,41,42,43,44,45,46,47,48,49].

**Figure 4:**
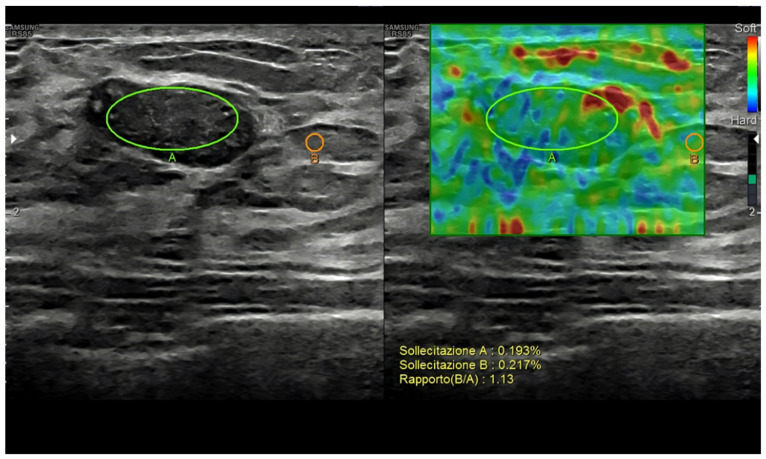
Breast fibroadenoma. Strain ratio allows a semi-quantitative assessment of the lesion-to-fat stiffness.

The use of elastography has been mostly focused on breast nodule characterization, but aspects such as tumor detection, tumor extent assessment, axillary lymph node status, percutaneous procedure guidance, and tumor response to treatment assessment must also be considered [41,50,51,52]. As a general rule, stiffer nodules (i.e., score 1 or 2, low strain ratio, low shear-wave speed), opposing a significant resistance to the changes, are thought to be malignant, while elastic ones (i.e., score 4 or 5, high strain ratio, high shear-wave speed) are usually categorized as benign [53] (Figure 4).

Breast nodules may, however, show an intermediate behavior (score 3), with overlapping findings and possible false positives (fibrotic and calcified fibroadenomas, sclerosing adenosis, radial scar, steatonecrosis, etc.) and false negatives (e.g., lymphomas, in situ ductal carcinomas, and small or low-grade or tubular invasive carcinomas) [54,55]. Elastography may increase US specificity, although this may come at the cost of decreased sensitivity. Consequently, images’ interpretation should never rely on elastography alone and should always be correlated with the grayscale appearance [53,56].

### 2.5. Contrast-Enhanced Ultrasound

As for Doppler vascular signal, the intensity of contrast medium enhancement represents an indirect indicator of tumor microvascular density, correlating with a malignant nature and with the cancer grade. First introduced to boost the low-sensitivity Doppler systems of the 1990s, microbubble contrast media are now employed during real-time, low mechanical-index, grayscale US. However, despite a high number of publications, contrast-enhanced US (CEUS) has not significantly impacted breast imaging. This is mostly due to the kind of contrast media available until now, which work better at the low frequencies employed to scan the abdomen than at the higher frequencies needed to image superficial structures such as the breast [57,58,59,60,61,62].

Malignant breast nodules show a quick and strong but transient enhancement, with variable degrees of heterogeneity [63,64,65,66,67]. Irregular, tortuous, radially distributed vessels are seen around and inside tumors [35]. A time–intensity curve can be obtained to quantify the microbubbles’ behavior over time. Three-dimensional images may better render the tumor angioarchitecture [68]. CEUS may measure the tumor size better than conventional B-mode US. Additionally, it may assess the breast tumor response to neoadjuvant treatment, showing 87% aggregated sensitivity and 84% aggregated specificity in a recent meta-analysis [69]. Tissue harmonic imaging (THI) and contrast harmonic imaging (CHI) also have value in the detection and characterization of breast tumors.

### 2.6. Three-Dimensional Ultrasound

**Figure 5:**
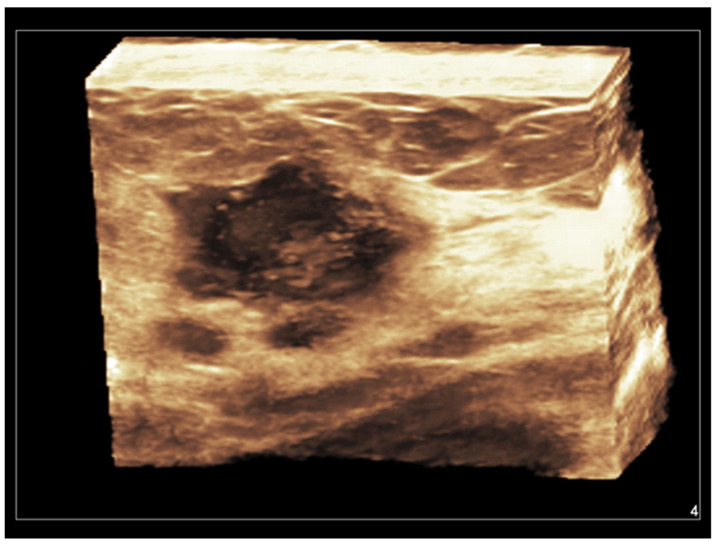
Invasive ductal carcinoma of the breast. Coronal 3D display of infiltrating tumor margins.

Both hand-held and automated linear transducers are available for use in high-resolution 3D breast imaging. Three-dimensional US, also called volumetric US, allows for obtaining a surface rendering of normal and abnormal breast structures. With a single pass of the ultrasound beam, a 3D reconstructed image is formed in the coronal, sagittal, and transverse planes, allowing a more accurate assessment of anatomical structures and tumor margins [11]. Coronal images, being parallel to the skin, represent a unique opportunity allowed from 3D US. A vivid representation of breast tumors can be obtained, with a retraction, star-like profile strongly supporting the diagnosis of malignancy [70,71,72,73,74] (Figure 5).

Additionally, lesions can be automatically or semi-automatically delimited on the three planes, and their volumes can be quantified. Nodules’ growth and cancer regression during treatment can be objectively evaluated through serial scans. Three-dimensional US can be combined with harmonic imaging, Doppler techniques, and CEUS [16]. Additionally, percutaneous procedures can be advantaged by viewing multiplanar and three-dimensional images.

### 2.7. MicroPure

**Figure 6:**
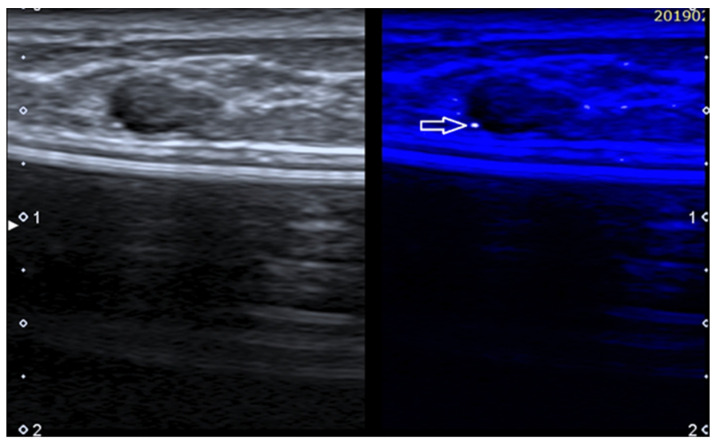
Fibroadenoma in a patient with history of augmentation mammoplasty. MicroPure allows to easily detect a bright dot (arrow) due to a small calcification.

Grouped microcalcifications represent a very important finding in breast imaging and a significant US limitation, being hard or impossible to detect, particularly if not associated with a nodule [75]. MicroPure^TM^ is a software from Canon (Tokyo, Japan) that allows to highlight microcalcifications (Figure 6).

MicroPure combines non-linear imaging with speckle suppression, extracting the calcification from the heterogeneous background. Filtered microcalcifications are shown as bright dots inside a dark blue background overimposed to a grayscale US.

### 2.8. Automated Breast Ultrasound

So-called “manual” or “hand-held” US is limited by operator dependence, non-reproducibility, and the inability to image extensive breast areas and store volumes [64]. To overcome these limitations, an automated breast US (ABUS) system, or automated breast volume US, has been developed. ABUS allows a technologist to simultaneously acquire large volumes of tissue, from the skin to the chest wall, by using a large, specific transducer (15 cm) [76,77]. These volumes, consisting of a high number of thin scans, are stored to be visualized and reformatted by a doctor at another moment or another place. Coronal views are of special value [78]. By now, ABUS has been considered an adjunct to mammography to screen patients with dense breasts. ABUS has a shown good agreement with manual US in terms of detection rate and BI-RADS categorization [79,80,81]. Architectural distortions and peritumoral infiltrations may be better displayed than with manual US, while large masses can be easily measured [82]. A good correlation with MRI in the assessment of tumor response to treatment has been demonstrated [81]. ABUS is less time-consuming than manual US and causes less fatigue to the operator [64]. It should be considered, however, that any probe-mediated palpation finding is missed with ABUS and that the direct interaction between the patient and the physician is completely lost.

### 2.9. Computer-Assisted Diagnosis—S-Detect

Owing to deep learning algorithms, artificial intelligence systems are able to automatically detect and quantify a number of features from US images [16,68,83,84,85,86,87,88,89,90]. This may allow for the simple and reproducible detection and characterization of breast lesions as well as the prediction of the response to treatment in patients with locally advanced breast cancer. Computer-assisted diagnosis (CAD) can be employed as a second reader to improve the accuracy of the operator in US or CEUS imaging of the breast [64,91,92,93]. The software analyzes the targets identified by the operator, showing their shape and their risk of malignancy based on the BI-RADS lexicon or other descriptors. A CAD system works through four successive phases: pre-processing, segmentation, feature extraction and selection, and classification [64,94]. The analysis can be approved or rejected by the operator [95,96].

**Figure 7:**
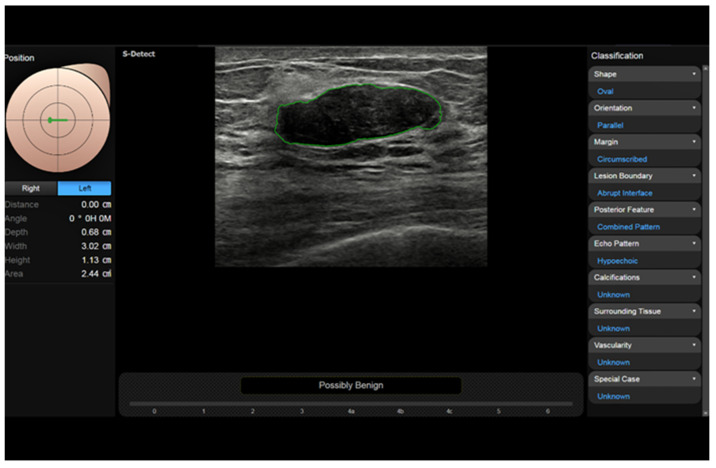
S-Detect automatic measurement and categorization of a breast nodule (fibroadenoma) using BI-RADS descriptors.

CAD systems can be offline, located in a personal computer, or inserted directly on the scanner. An example of the latter is represented by S-Detect^TM^, a semi-automatic tool from Samsung (Busan, Republic of Korea) [97,98,99,100]. The operator manually places a marker inside a lesion, and then, the software traces the border (adjustable) and analyzes and classifies the lesion according to the US descriptors from the BI-RADS (Figure 7).

In a study on the differential diagnosis of breast lesions, the sensitivity of five operators of different experience levels was >90% and specificity was 50–75%, while S-Detect had 90% sensitivity and 71% specificity [101]. Advanced systems are fully automatic, are based on convolutional neural networks, and have a high capability of recognizing the images.

### 2.10. Ultrasound Nomograms

Radiomics can extract many quantitative features from US images through a computer algorithm [102]. To create a nomogram, first, well-established US features are selected and extracted by the training experts; then a model is built; and finally, the model is tested, possibly using both an internal and an external validation cohort of patients. The quantitative features extracted through computerized algorithms can be employed for the differential diagnosis of breast abnormalities or to predict the prognosis (risk of lymph-node metastasis, risk of high nodal burden, etc.) or establish in advance the response to treatment of breast tumors [103].

### 2.11. Images Fusion and Virtual Navigation

Fusion imaging consists of merging digital images from two different modalities to improve the overall performance, with special reference to lesions’ localization at second-look US and to the percutaneous approach to those abnormalities poorly visible with US. Real-time images of the US system are combined (directly overimpressed or shown synchronized side by side) with those previously uploaded from a previous mammography, CT, MRI, or PET exam. This allows real-time, virtual navigation through the volume. The structures invisible under US but visible with other modalities can be operated using US-guided biopsy navigated by the other modality [64]. The indirect systems employ artificial skin markers placed before the exam, while the direct systems use some anatomical landmarks as spatial references [104,105,106]. Algorithms have been developed for assessing organ motion induced by breathing and movement [64].

BreastNav^®^ is an interesting system from Esaote (Genoa, Italy) allowing fusion between a prone MR scan and a supine US scan through 2D remodeling applied to the breast from an anatomical landmark established during the MR scans. An electromagnetic sensor is applied to the transducer. This allows a targeted second-look US assessment of findings not determined at MRI. The US fusion volume navigation technique can be used to scan the breast nodules requiring follow-up [64].

Needle-tracking software facilities, based on specific sensors, allow to simulate the needle path during US-guided percutaneous procedures. This tool may increase effectiveness and safety.

## 3. Changing Clinical Scenarios—The Current Impact of US in Breast Practice

### 3.1. Primary Ultrasound

There are a good number of clinical settings where whole-breast, bilateral, axilla-including US must be regarded as the first imaging modality to be employed, after physical examination. In many circumstances, US will be conclusive to solve the issue, while in others, US results will prompt further investigation.

A palpable breast mass or swelling always requires imaging assessment, and US will effectively differentiate cysts (BI-RADS 2), non-suspected nodules (BI-RADS 3), and suspected nodules (BI-RADS 4 and 5) [11,14,107,108,109].

Dense breasts in young women cannot be imaged efficaciously with mammography. However, more and more women nowadays are asking for an imaging assessment to feel themselves followed and to reduce their anxiety. These subjects can be asymptomatic or may present with symptoms such as breast pain or tenderness. US has become the supplemental screening tool of choice for cancer detection in women with dense breast tissue.

MRI is the most effective tool in measuring the primary breast tumor extent, and it allows the detection of additional foci of mammographically and/or sonographically occult disease in women with newly diagnosed cancer. However, US is also useful for preoperative breast cancer staging, with special reference to axillary lymph nodes’ status, allowing to perform examination of the whole axilla with level 1–3 lymph nodes.

Nipple discharge, a common circumstance of special relevance if bloody or dark, can now be imaged with US. Galactography is no longer employed, and MRI is reserved to selected cases.

**Figure 8:**
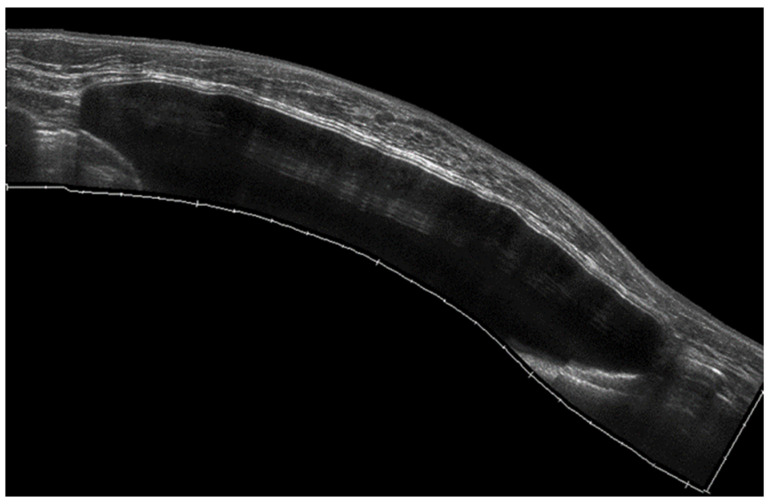
Sub-muscular breast implant as displayed using an extended FOV acquisition.

US works well for newborns, children, and adolescents with breast abnormalities (grading of premature thelarche, etc.). Breast disorders in pregnancy should be studied using US, at least because of radioprotection needs [11]. Lactating breasts are also difficult to image with mammography because of the glandular density, and US is the primary imaging modality in women with abnormal symptoms during lactation [11]. Male breast abnormalities should be studied with US, which can easily differentiate gynecomastia from cancer. Breast assessment in women scheduled for augmentation surgery or for hormonal therapy because of infertility is also based on US, particularly for younger women. Periodic assessment after mastoplasty is carried out with US, while MRI is employed when an implant rupture is suspected [110] (Figure 8).

Owing mainly to the real-time characteristic, US is the method of choice to guide diagnostic and therapeutic percutaneous procedures whenever the target is adequately visible with US [111,112,113,114]. US can also be useful during treatment planning for breast radiation therapy [11]. Intraoperative US can guide the surgeon towards a quicker and more effective approach at the operating table, decreasing the incidence of positive margins and the consequent need for re-excision [11].

**Figure 9:**
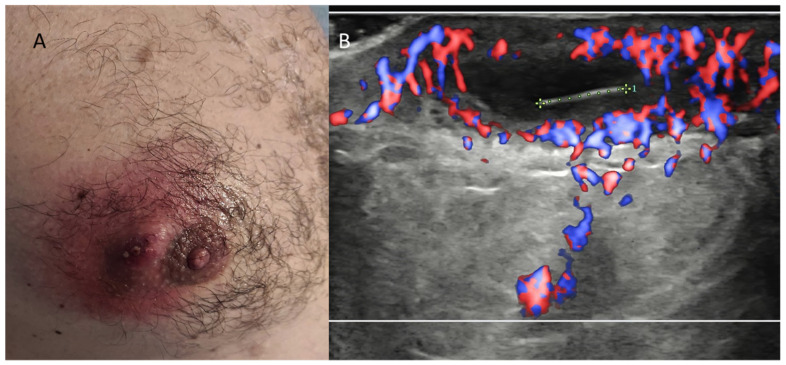
Ingrown areolar hair causing abscess formation in a young male. (**A**) Clinical photograph. (**B**) Power Doppler US imaging. The 6-mm linear echoic hair and the surrounding strong hyperemia are readily recognizable, allowing a confident differential diagnosis with Montgomery glands inflammation.

Though focused on the mammary parenchyma, breast US has the ability to assess all the tissue layers, from superficial to the gland (dermis and hypodermis) and deeper to the gland (retro-glandular fat, chest wall, and pleuro-pulmonary area) (Figure 9).

Patients presenting to a US exam for abnormalities arising from these areas, as well as non-glandular incidental findings during breast US, can be readily evaluated. Second-level imaging modalities are employed in selected cases [14,33,107,108,109,110,111,112,113,114,115,116,117,118].

### 3.2. Complementary Ultrasound

**Figure 10:**
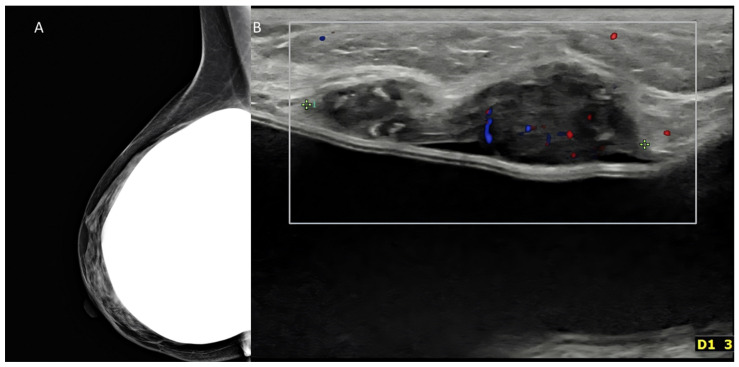
Palpable mass in a woman with history of augmentation mammoplasty. (**A**) Mammography oblique digital view was negative in this case, despite use of tomosynthesis. (**B**) US could detect a nodule immediately above the implant. The tumor proved to be a triple-negative invasive ductal carcinoma at surgery.

US is both an adjunct and a complement to mammography (Figure 10).

Screening of asymptomatic women is based on mammography. However, younger women are now asking to be imaged for an early diagnosis of breast cancer. In this setting, mammography alone is not enough, and combination with US, or with ABUS, is mandatory [115,116,117,118,119].

Surveillance of women at high risk, bearing a hereditary/familial risk, is currently performed with contrast-enhanced MRI. However, US can represent a useful adjunct, at least to increase the time interval between each MRI exam. US complements initial mammography and, if needed, MRI in the assessment of local and regional tumor extent. MRI is the gold standard in the assessment of response to treatment both in patients with neoadjuvant therapy for a locally advanced breast cancer and in patients with a metastatic breast cancer. However, being an easily repeatable exam, US can be performed serially and allows multiple measurements of the size changes. US complements annual mammography in the loco-regional follow-up of patients with a history of breast cancer [120,121].

### 3.3. Second-Look Ultrasound

US is frequently used as a targeted, second-look option for patients imaged with other breast imaging modalities.

Since the beginning, US has been employed to better define any abnormality found with mammography. US is employed to differentiate cystic and solid opacities, to assess the level of suspicion of any nodule, and to assess distortion or any other changes. US is performed immediately after a mammography requiring further work-up or during patient recall. Finally, US is employed in patients with a palpable abnormality and negative mammograms.

Contrast-enhanced MRI may require a targeted US scan in the case of enhancing or apparently enhancing focal changes or when an intense background parenchymal enhancement may mimic or masquerade a breast nodule [82,122,123]. Lesions detected on MRI are often mammographically occult, but many of them can be detected with targeted US [11]. Lesions found with MRI can be located by US and, consequently, can be histologically clarified by US-guided biopsy [64].

Breast uptakes with a whole-body PET scan can also be further investigated with US to avoid false positive diagnoses. This applies to patients undergoing a PET exam because of breast cancer but also to subjects with non-mammary primary tumors during their staging or follow-up with molecular imaging.

**Figure 11:**
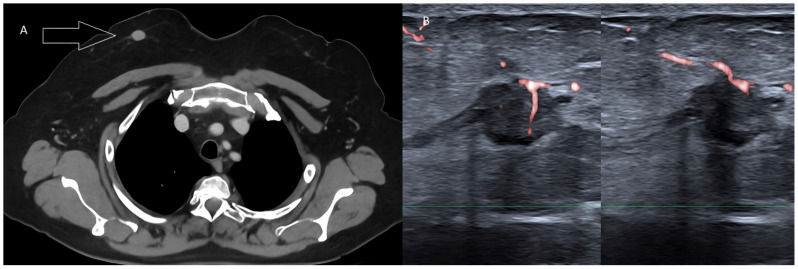
Pure tubular carcinoma detected incidentally during whole-body CT in a female patient with rectal cancer. (**A**) Contrast-enhanced, venous-phase CT scan detecting a nodule within the right breast (arrow). (**B**) Targeted US scan displaying the malignant lesion.

Breast nodules are frequently detected during contrast-enhanced or unenhanced CT exams, chest scans, abdominal scans, or whole-body scans (Figure 11).

US works well as a quick and simple tool to confirm or rule out a nodule and to establish the need for further investigation or for patient follow-up.

## 4. Conclusions—Not Everything That Glitters Is Gold

Despite the tremendous advances illustrated in this review, it is important to highlight a number of ongoing limitations and pitfalls. A large, fatty breast is still a problem for US scan, and US should never be employed as the only modality to evaluate such a case [11]. Microcalcification must be accurately detected and characterized, and this aspect is still a duty of mammography as US has limited sensitivity, especially when microcalcifications are not located inside a nodule. Moreover, a careful, multiparametric, US exam of the whole breasts and axillary cavities is time-consuming. Both in the case of sonographer-performed US and physician-performed US, this examination requires an adequate amount of time. Breast US should be carried out with top-level scanners, equipped with all the software capabilities allowing to perform a multiparametric investigation. Due to continuous direct contact with the patient and the high emotional involvement of women with any breast-related trouble, mammary US requires special abilities from the operator to concentrate and, at the same time, show good empathy [124,125,126,127]. Additionally, despite many attempts at methodological and lexical standardization and the introduction of ABUS, US is still a subjective exam that depends on the skills of the single operator, with limitations in terms of objectivity and intra-observer and inter-observer reproducibility.

Finally, it must be considered that competition from other imaging modalities is strong. As with US, mammography and MRI have also shown significant improvements through the years, and other techniques are being proposed as well [128,129]. Digital tomosynthesis and contrast-enhanced spectral mammography are quite important options, now employed routinely [82,98,130,131,132]. Quantitative perfusion MRI, MR lymphography, blood oxygenation level-dependent MRI, and diffusion-weighted MRI have all increased the impact of this modality [130,131,132,133,134]. However, it should always be kept in mind that all imaging modalities have their points of strength and weakness. Consequently, all modalities must be employed in the most appropriate way to cover the diagnostic needs of each single patient.

## Data Availability

All data are reported in the manuscript.
